# The Emergence of the Old Drug Captagon as a New Illicit Drug: A Narrative Review

**DOI:** 10.7759/cureus.55053

**Published:** 2024-02-27

**Authors:** Joseph Pergolizzi Jr, Jo Ann K LeQuang, Eugene Vortsman, Peter Magnusson, Salah N EL-Tallawy, Morgan Wagner, Rania Salah, Giustino Varrassi

**Affiliations:** 1 Pain Medicine, NEMA Research, Inc., Naples, USA; 2 Scientific Communications, NEMA Research, Inc., Naples, USA; 3 Opiate Task Force, Northwell Health, Long Island Jewish Medical Center, New York, USA; 4 School of Medical Sciences, Örebro University, Örebro, SWE; 5 Anesthesia and Pain Management, Faculty of Medicine Minia University and National Cancer Institute, Cairo University, Cairo, EGY; 6 Anesthesia, King Khalid University Hospital, College of Medicine, King Saud University, Riyadh, SAU; 7 Entrepreneur Program, NEMA Research, Inc., Naples, USA; 8 Medicine, Alfaisal University College of Medicine, Riyadh, SAU; 9 Pain Medicine, Paolo Procacci Foundation, Rome, ITA

**Keywords:** amphetamine abuse, drug of war, fenethylline, captagon (fenethylline) dependence, captagon

## Abstract

First developed in the 1960s in Europe and approved briefly for use in the United States, fenethylline (sold as Captagon, one of its early trade names) is now a prominent drug of abuse in the Eastern Mediterranean Region. The drug was withdrawn from the United States market because of side effects that included hallucinations, visual distortions, and psychosis; it has also been linked to rare cases of myocardial infarction, seizures, and delusions. The chemical synthesis of fenethylline is straightforward and inexpensive. Manufactured in clandestine labs in Southern Europe and the Middle East, these amphetamines had been used by affluent Middle Eastern young people for recreation or study aids. Captagon has periodically emerged as a drug used in combat and conflict, and it was implicated in the 2015 riots in Paris. It has been described as “chemical courage” for combatants giving them focus, energy, and endurance in battle situations. Captagon is addictive but no cases of direct captagon-associated mortality have been reported. The use of drugs in war is nothing new, but captagon is also used heavily in the civilian population in war-torn areas to help them cope with food insecurity and maintain courage in dangerous situations. Captagon production and distribution drives the Syrian economy, but the drug’s use is limited to certain regions and is rarely seen in North America. The drug is available online, but product may be contaminated with the inclusion of procaine, caffeine, or other substances.

## Introduction and background

Fenethylline, also known as captagon, was synthesized in Germany in 1961 as part of a broader program to explore the cardiovascular and central nervous system effects of theophylline derivatives [1]. It was initially marketed in Europe as an over-the-counter product that substituted for amphetamines, but quickly was relegated to prescription-only status [2]. The commercial marketing of Captagon mainly occurred in Europe and the Middle East in the 1960s, when the drug was indicated for attention deficit disorder, narcolepsy, and as a central nervous system stimulant. These early products were offered as tablets containing 50 mg of fenethylline, which in the body metabolized to amphetamine and theophylline. The pharmacologic effects of the drug are considered to be the actions of these two metabolites, of which the main one, amphetamine, can be considered a central nervous system stimulator. Fenethylline was not approved for any specific medical indications and was moved to Schedule I controlled substance in the United States in 1986 [3]. While the drug rapidly fell out of favor because of its associations with hallucinations, aggression, and dependence issues, France, Belgium, Germany, Luxemburg, and the Netherlands allowed it to be sold legally up until around 2013 as a treatment for narcolepsy [4]. Marketed under several trade names, the best known of which was Captagon, the drug today is mainly called captagon after its original brand.

The first seizure of illicitly trafficked captagon occurred in Germany in 1984 [2]. Captagon has also been used by European athletes; while it does not enhance athletic performance, it increases physical endurance and its use is considered a form of doping [5]. Captagon use by street drug users in Germany increased at various times in the 1980s when cocaine and other stimulant drugs were in short supply or highly priced [2,6]. While captagon is today manufactured in clandestine laboratories in Southern Europe and Turkey [3], it is emerging as both a recreational drug of abuse as well as a drug associated with war in the Middle East. In response to market locations, the illicit manufacture is migrating toward the Middle East [3]. While captagon’s manufacture, use, abuse, and trafficking remain concentrated in the Middle East at present, substance use disorder is a global problem and it is not clear if captagon may have a global impact. Captagon, related to amphetamines, enhances dopamine production and thus may promote feelings of well-being, pleasure, and even euphoria [7]. It is an inexpensive and addictive substance that may be used to sharpen concentration, promote feelings of well-being, and increase stamina. This is a narrative review of captagon, its current place in the illicit drug market, and future considerations for this agent.

The authors searched the PubMed database in December 2023 for the keyword “captagon” and obtained 60 results. No limitations were used and many of these articles were older and less relevant to our research interests; only 20 were published in the last decade. The nature of the subject was such that authoritative websites, newspaper articles, journal articles, and online searches were used to supplement the limited information in the scholarly literature. The dark web was not searched.

## Review

Captagon production and use remains geographically concentrated in the Eastern Mediterranean Region (EMR), which is now facing an unprecedented drug crisis in addition to ongoing geopolitical struggles; see Table 1. The drugs of abuse used in the EMR differ from the drugs abused in Western Europe and the United States. The EMR is defined by the World Health Organization (WHO) as encompassing 22 members with about 700 million inhabitants [8], many of these countries are facing increasing rates of use of other illicit substances, including but not limited to the use of captagon [9]. The EMR is a vast and heterogeneous region, home to economically devastated nations, such as Yemen and Somalia, as well as some of the wealthiest nations on earth, such as the United Arab Emirates and Qatar. History, culture, and recent political events also vary widely among these nations. While most of these nations are historically Muslim, the cultures of these countries vary as do their drug laws. Substance use disorder and illicit captagon are emerging as a regional issue at this time with more widespread and even global implications [10]. 

**Table 1:** The Eastern Mediterranean Region (EMR) is large, diverse, and dealing with problems of abuse of various illicit drugs in different ways. The nations of the EMR face challenges with illicit drug use [8,11-40]

Nation	Population	Note	Main illicit substance(s) used
Afghanistan	40.1M	50% <15 years of age	Hashish and 12.5% of population uses opium with increasing use by women and children.
Bahrain	1.5M	Archipelago, smallest nation in Middle East	Synthetic drugs, methamphetamines, heroin. Death penalty for drug sales. About half of those arrested on drug charges are foreign.
Djibouti	1.1M	Somalians are largest ethnic group	Khat (also spelled qat) is a natural but addictive substance and is legal and sold by the state. It is widely used, especially by men, but is expensive. Khat is illegal in many other countries.
Egypt	109.3M	Illicit drug culture centers around Cairo	Hashish, opium, cannabis (called bango), tranquilizers, also widespread use of tramadol (weak opioid). Highest heroin consumption in North Africa.
Iran	88.0M	Shia Muslim majority	Opioids, including heroin, crystal meth, hashish with drug use increasing. Opium imported from Afghanistan drives high opium use. The once-large gap between male and female drug users is narrowing. Harm reduction programs are in place along with progressive approaches to substance use disorder rehabilitation.
Iraq	43.5M	Population increasing rapidly	Synthetic stimulants, hashish, prescription tramadol, opium, heroin, cannabis, benzodiazepines. Drug use is increasing, especially among youth and women.
Jordan	11.2M	Drug use has increased rapidly in last 10 years	Synthetic stimulants, crystal meth, hashish, opium, tranquilizers. Use of captagon described as epidemic. Most frequent crime in Jordan is a drug-related offense.
Kuwait	4.3M	About 70% are expatriates with no straightforward path to citizenship	Opioids, synthetic stimulants, crystal meth, cannabis. Drug sale is a death-penalty offense. The use of gabapentin or pregabalin without prescription may subject the user to up to five years in prison.
Lebanon	5.6M	About two-thirds Muslim, one-third Christian	Tranquilizers, hashish, cannabis, cocaine, heroin.
Libya	6.7M	82% of population is urban	Hashish, heroin, and cocaine used and trafficked; major “transit route” for drugs. Drug trafficking has increased since the death of Gadhafi. Drug prices are “cheaper than food”.
Morocco	37.1M	Only monarchy in North Africa	Medical marijuana since 2021 but recreational use illegal. Cocaine and opioid use is rare but data are sparse. Mandatory treatment for drug offenders is a recent development.
Oman	4.5M	46% are immigrants	Hashish, heroin are most popular but still relatively rare. Growing use of synthetic stimulants. Methadone, once used to treat heroin use disorder, was outlawed in the 1990s.
Pakistan	231.4M	World’s 5^th^ most populous nation	Cannabis and heroin are cheap and widely used, mostly from Afghanistan. Charas is a local drug product made from concentrated cannabis resin. Prescription drug diversion occurs. Enforcement of drug laws is increasing.
Palestine	4.9M	Conflict area	Despite social stigma, tramadol, opioids, cocaine, cannabis, and alcohol are widely used. Captagon used by young people. Polysubstance abuse reported.
Qatar	2.7M	In terms of per capita GDP, richest nation on earth	Substance use disorders are main cause of disability, despite zero-tolerance laws. Many prescription and over-the-counter drugs familiar to the West are banned here, including ibuprofen and hormone replacement therapy.
Saudi Arabia	36.0M	Monarchy and 12^th^ largest nation on earth	Drug use increasing, particularly synthetic substances such as “Shabu” plus cocaine and opioids. 40% of drug users report using captagon, sometimes as a study aid.
Somalia	17.1M	War-torn nation devasted by poverty	Khat is legal and widely used. Hashish and opioids, particularly tramadol, becoming more common. More men than women use drugs, but drug use in women is increasing.
Sudan	41.8M	3^rd^ largest nation in Africa by population and land area	Cannabis is most frequently abused but tramadol, benzodiazepines, cough syrups, and anticonvulsant use is increasing. Amphetamine use is less frequent, likely due to cost.
Syrian Arab Republic	21.3M	Emerging narco-state	Synthetic stimulants, most notably captagon. In 2022, over $5B in illicit captagon from Syria was seized in other nations.
Tunisia	12.3M	98% Muslim with equal rights for men and women	Cannabis, buprenorphine, more limited use of heroin. Harsh penalties for use, consumption, or sale of drugs even for first-time offenders.
United Arab Emirates	9.4M	Federation of 7 emirates	Drug use is minimal by international standards although hashish and heroin are used and there is some prescription drug diversion. Zero-tolerance laws and harsh penalties for drug use and trafficking.
Yemen	33.0M	Mainly Arab, tribal populations, small Jewish population	Khat is legal and used by ~90% of men and ~25% of women, and is culturally accepted. Khat dulls the appetite and helps families manage life with food insecurity. Hashish, cocaine, and amphetamines are used but data are limited.

Historically, the EMR has been a major exporter of opium, but economic shifts, political turbulence, wars, and population displacements have altered patterns of consumption of illicit substances [9]. Captagon has not been legally produced anywhere in the world since 1986, but illicit captagon is widely sold in many EMR nations, including Iran, Pakistan, and other nations; in Saudi Arabia, captagon use is more frequently the cause of hospital admission than opioid abuse [9]. Globally, about a third of the amphetamine products seized in a year are captagon tablets, typically confiscated in the EMR nations [2]. The most alarming of the emerging trends with captagon occurs in Syria where the drug is thought to improve military readiness by virtue of giving users stamina, alertness, and focus [7]. Stimulant drugs have often been used in combat, but this important subject has not been well studied [41].

History of captagon

Captagon is a psychostimulant agent, a codrug of amphetamine and theophylline, with the generic name of fenethylline [3]. When it was commercially marketed, the brand names of Captagon, Biocapton, and Fitton were used, but today the illicit drug is universally called captagon [3]. Its chemical name, fenethylline or (R,S)-1,3-dimethyl-7-[2-(1-phenylpropan-2-ylamino)ethyl]purine-2,6 dione, defines an amphetamine conjugated with theophylline using an alcohol chain [2]. It is usually produced as a hydrochloric salt with a molecular weight of 377.95 g/mol [2]. In the United States in the 1960s and 1970s, it was indicated for the treatment of pediatric attention deficit disorder, narcolepsy, and depression until it was removed from the market [3].

**Figure 1:**
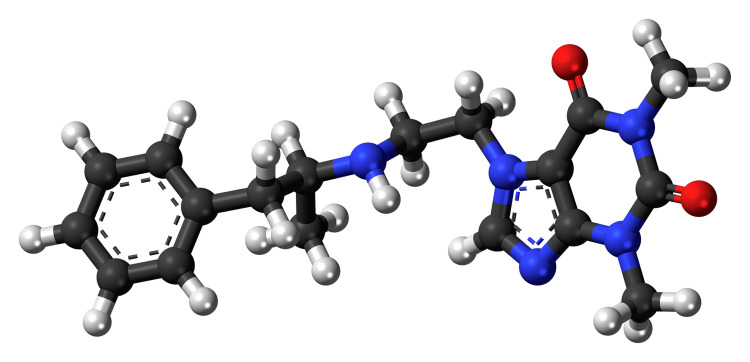
Fenethylline (captagon) molecule. Black is carbon, White is hydrogen, Red is oxygen, Blue is nitrogen Molecule drawing by Jynto, created with the Discovery Studio Visualizer, CC0 via Wikimedia Commons.

Chemically synthesizing fenethylline is relatively simple and the raw materials necessary for the task are available for legal purchase [2,3]. Manufacture is relatively straightforward and inexpensive [3]. Once thought to be mainly manufactured in clandestine labs in the southeastern part of Europe, it appears that manufacturing efforts have migrated to the Middle East and Northern Africa [3]. Captagon is available on the street in many locations and online without a prescription, especially from sites on the dark web [3]. Clandestine production of the drug has resulted in contaminated products with detectable amounts of adulterants, such as procaine, quinine, caffeine, and metronidazole [3]. The molecule appears in Figure 1.

Metabolism and toxicology

There is a paucity of information in the literature on captagon metabolism and toxicology [2]. The primary metabolite of captagon is amphetamine, a central nervous system stimulant. Amphetamine acts as an agonist at the trace amine-associated receptor 1 (TAAR-1), which boosts dopamine signaling. This dopamine effect may produce behavioral changes such as irritability and aggression and can lead to dependence [7]. Captagon metabolizes to amphetamine (24.5% of dose per oral) and theophylline (13.7% of dose per oral). Theophylline, a xanthine agent, is a weaker stimulant on the order of caffeine with a narrow therapeutic window; at supratherapeutic doses it is associated with arrhythmias and gastrointestinal side effects [42,43]. Captagon has greater lipophilicity than either theophylline or amphetamine alone, allowing its more rapid absorption into the central nervous system, but amphetamine penetrates brain tissue more rapidly than captagon [44]. Theophylline is metabolized via cytochrome enzyme P-2D6 (CYP2D6), while amphetamine inhibits CYP2D6. For that reason, amphetamine is eliminated more rapidly from the body than theophylline, but these two agents act synergistically with each other to enhance the individual drugs’ psychoactive properties [7].

Captagon is available in the form of oral tablets but these can be crushed, heated, and injected intravenously for a more powerful and immediate effect [3].

Safety and side effects

Safety concerns caused the withdrawal of this product from the commercial market. The most concerning side effects of captagon include psychosis, visual hallucinations, visual distortions, acute heart failure, seizures, and acute myocardial infarction [45]. The pathophysiology of amphetamine-induced myocardial infarction has not been elucidated but it is likely associated with vasoconstriction and destabilization of the thrombus caused by the amphetamine [46]. Other side effects are tachycardia, increased body temperature, and rapid respiration. Over time, captagon use can result in sleep deprivation, lethargy, and depression. Some long-term users of captagon may develop malnutrition, as the drug can depress appetite and reduce interest in food [47]. However, prolonged wakefulness and reduced appetite may be beneficial for combatants in times of war. Hallucinations and psychosis have been reported with captagon use along with depression, irritability, and aggression [3]. It is unclear whether captagon or other amphetamines lead to psychosis in general or whether they provoke prolonged sleeplessness, which, in turn, triggers the psychosis [48,49].

While insomnia is a common side effect of prolonged exposure to captagon, the effect of captagon on sleep architecture has not been well studied. In a survey of 78 male patients with diagnosed amphetamine-induced psychosis, the majority of participants (92.9%) experienced insomnia while taking captagon, but insomnia was often intermittent [48]. The longest period of persistent wakefulness observed in this study was seven days and occurred in one participant [48]. Most participants experienced some degree of insomnia, and 61% self-treated this condition with some sort of sleep aid, including quetiapine, clozapine, benzodiazepines, antihistamines, alcohol, or marijuana [48]. Subjects in this study reported that psychotic symptoms and/or hallucinations were mitigated or ended completely when normal sleep was restored [48]. This observation has led to the notion that amphetamine-induced psychosis might be effectively treated with benzodiazepines, but there are currently no guidelines or expert consensus to support this [48].

Delusions may also occur with prolonged captagon use. In a study from Saudi Arabia of 101 men between the ages of 19 and 46 years being treated for captagon addiction, 25.7% developed delusions of infidelity. Compared to other patients, those with jealous delusions had a higher divorce rate [50]. It has been suggested that morbid jealousy may be related to captagon-induced disordered sleep [48]. In rare cases, captagon may provoke arrhythmias and there is a report in the literature of two cases in which captagon has induced changes visible on the electrocardiogram that appear similar to those of congenital Brugada syndrome [51].

Use and abuse

Captagon is an addictive substance, although it is considered less addictive than conventional amphetamine, perhaps because it penetrates the brain tissue more slowly than amphetamines [47]. Captagon is often part of polysubstance drug abuse. Polysubstance drug abuse may be intentional, opportunistic, or unintentional, when a user takes a contaminated product mixed with other substances. Polysubstance abuse is a global trend that occurs in the EMR as well as in other regions [9,52]. However polysubstance abuse in the EMR is more likely to include captagon in the mix than polysubstance cocktails in Western Europe and North America. Captagon plus an opioid is a common combination, including captagon plus methadone [9]. Other captagon combinations are its use with alcohol and/or cannabis [3].

There are reports of withdrawal symptoms upon drug discontinuation, most notably headache and depression [3]. There is no established protocol or guidance for navigating captagon discontinuation. Cultural pressures may stigmatize those taking captagon from seeking medical assistance to discontinue the drug. In a study of university students using captagon over several days, it was noted that even those who wanted to stop taking the drug hesitated to ask for help because of the shame attached to drug abuse [53].

Risk factors for captagon use

Captagon is illegal in all markets, and motivations for using this agent vary. A qualitative study of 10 Jordanian university students who used captagon reported that most students took the drug to manage personal and academic pressures. These students were able to obtain the drugs from friends and found that over time, captagon created more problems than it solved. Respondents reported that after using captagon for several days in a row, they became disorganized and forgetful [53]. It has been estimated that among Saudi Arabians with any type of substance use disorder, approximately 40% use captagon, whether alone or with other substances [3].

Most captagon users are young men [3]. There are no known cases of captagon directly causing mortality [54].

A drug of war

Captagon has been nicknamed “chemical courage” because of its use in military operations and riots, where it can increase endurance, heighten perceptions, and allow prolonged wakefulness [7]. Anecdotally, captagon has been reported to suppress pain, although the mechanisms behind this effect are not known [7]. Soldiers or others engaged in conflict may find their sense of invincibility enhanced by captagon, including boosting a general sense of well-being and fearlessness. It may be that this sense of being nearly invincible reduces or blocks pain perception. Captagon is reported to be taken not only by soldiers, but also by civilians in war-torn areas to help them manage desperate and terrifying situations [55].

While drug use in war is nothing new, captagon may represent an important evolution in what has been called pharmaco-terrorism [56]. The question arises as to whether captagon is taken to allow trained soldiers to carry out military operations or whether these drugs may promote or encourage violence among civilians or paramilitary troops [3]. Under Sharia law observed by most Muslim states, Muslims are not to use any drugs of abuse nor may they consume alcohol; an exception is made for drugs serving a defined medical purpose. While Al-Qaeda observes this restriction and thus bans the use of captagon even by the military in times of war, the Islamic State in Iraq and Levant (ISIL), formerly known as ISIS, allows the use of captagon in warfare [3,55,57].

The 2015 riots in Paris were thought to involve widespread use of captagon and other psychoactive substances [58].

The economics of captagon

Syria has been most severely impacted by captagon, because its economy is now dependent to a large degree on captagon production and distribution. It is estimated that 90% of the foreign currency coming into Syria is a result of the captagon trade [59]. Captagon sales serve two distinct and lucrative markets: combatants and those living in conflict zones, on the one hand, and affluent young men in the EMR, on the other hand. It has also been reported to be taken by some individuals in the EMR as a weight-loss drug [59].

Since 2010, the Syrian “conflict” has resulted in over 200,000 deaths and 12 million displaced persons [55]. While there are numerous political, social, economic, and religious reasons for this conflict, most reports and academic observers overlook the potential role that captagon may have played [55]. And since the Syrian economy is now dependent on captagon sales, its ability to control and rein in the use of captagon is limited [55]. Syria has been made both wealthy and economically vulnerable by illicit captagon production and distribution. Continued dependence on its production may impact the ongoing civil strife in Syria and other regions of the EMR.

Counterfeit captagon

Analysis has found that many samples of captagon are mixtures of amphetamine plus caffeine, rather than fenethylline hydrochloride [60]. In some cases, methamphetamine rather than amphetamine is found in counterfeit captagon. Other substances that have been found in these pills include ephedrine, chloroquine, quinine, theophylline, and acetaminophen [61]. One “captagon” seizure in London in 2016 found pills that contained only caffeine. Black market captagon can contain highly variable amounts of amphetamine, from trace amounts to substantial quantities [4].

Discussion

Captagon is not widely produced, distributed, or sold in the United States; in fact many American drug users have never even heard of this substance. The emergence of captagon as a global drug of abuse and its simultaneous lack of intrusion into the United States is an interesting occurrence. Looking globally at drugs of abuse paints an alarming picture of a multiplicity of agents which may be localized to one region or have the potential to spread to other parts of the world.

Captagon is a less addictive substance but it may be used in dangerous ways by rioters, combatants, and the military [58]. The use of drugs to propel demonstrations into street battles has been termed “pharmaco-terrorism” and represents a potential threat, particularly to urban environments prone to political and social demonstrations [58]. 

Driving the use and proliferation of captagon is the Syrian economy, which is largely dependent on the drug. Syria came to the business of illicit captagon in an unusual way because scientists and researchers in Bulgaria had established academic ties to Syrian colleagues. When captagon was made illegal in the 1980s, Eastern Europe and particularly Bulgaria remained the main producers of illicit captagon, for which there was still a small illegal market [62]. The unrelated intellectual exchange between pharmaceutical chemists and other researchers in Bulgaria and Syria led to cooperation in terms of setting up labs for Syria to manufacture illicit captagon [54]. This knowledge transfer enabled Syria to be able to manufacture captagon and similar amphetamine-type drugs, a boon for the fragile economy of a war-ravaged nation. While there was a potential market of affluent buyers for captagon in the United Arab Emirates and Saudi Arabia, the laws and strict penalties for illicit drug manufacture in those nations made production in those states ill-advised. Thus, Syria became the home of small clandestine facilities producing captagon, which could then be smuggled in small amounts into other nearby EMR nations [54]. The trafficking of captagon in some areas of the EMR is facilitated by high unemployment and depressed economic conditions. In fact, in some parts of the EMR, historic generations-old economic stagnation has made smuggling a long-standing business model; only the smuggled goods have changed, from cigarettes to hashish to fuel to weapons and now captagon [54].

Another reason for the popularity of captagon in the EMR may be due to the high proportion of guest worker populations in certain EMR nations. Guest workers are foreign nationals who come to EMR nations for economic opportunity but often find themselves working long hours or multiple jobs. For them, captagon can improve their stamina, enhance productivity, and allow them to manage sleep deficits and food insecurity. Part of the appeal of captagon in the EMR compared to, for example, methamphetamine or cocaine, is that captagon is considered a “milder” drug. Because captagon was once a legal product and even indicated for pediatric use, there is sometimes less stigma attached to its use than the use of marijuana, cocaine, or opioids [54]. 

The market value of Syria’s trade in captagon has been estimated at $3.5 billion, which may actually be an underestimation [63]. This has both bolstered and destabilized the Syrian economy, and it has negatively impacted the relationship Syria has with its neighboring states. More clinical research is needed to better understand this drug, particularly in terms of developing safe protocols for discontinuation.

This review has several limitations. It is a narrative review on a drug that has only been the subject of limited study. Where appropriate, we supplemented our research with websites, newspapers, and periodicals. Although the authors' interest in this drug is primarily clinical, the emergence of this drug in armed conflict has given it political nuances, which the authors found unhelpful and tried to avoid. Finally, the research involved a complex and heterogenous region, the EMR, and the authors did not wish to convey the impression that all nations had similar issues with illicit substances; they do not.

## Conclusions

Captagon is an illicitly produced amphetamine used widely in certain parts of the EMR but virtually unknown in the United States. This once-legal agent was taken off the market because of its association with hallucinations, visual distortions, and seizures as well as its addictive properties. While captagon is sometimes used as a study aid or weight-loss product, it is sold to affluent young people in the EMR mainly as a recreational drug. Unfortunately, either with recreational use or in combat, there are no or very limited data describing for safe discontinuation of the drug. Its more alarming use is the potential role it has or may continue to play in military activities. Captagon or “chemical courage” is thought to give combatants endurance, focus, and fearlessness in battle. While drugs have always played a role in warfare, it is not known if captagon may be a form of pharmaco-terrorism in that it might encourage violence rather than solely promote endurance and stamina. The ability to better research and regulate captagon as well as prevent its abuse is challenged by the fact that it is mostly produced in clandestine labs and distributed on the illicit market.
